# Voluntary Self-Exclusion and Contingency Management for the Treatment of Problematic and Harmful Gambling in the UK: An Exploratory Study

**DOI:** 10.3390/healthcare11192682

**Published:** 2023-10-04

**Authors:** Morgan B. Zolkwer, Simon Dymond, Bryan F. Singer

**Affiliations:** 1 School of Psychology, University of Sussex, Brighton BN1 9RH, UK; morgan.zolkwer.22@ucl.ac.uk; 2 Great Ormond Street Institute of Child Health, University College London, London WC1N 1EH, UK; 3 Mosaicism and Precision Medicine Laboratory, The Francis Crick Institute, London NW1 1AT, UK; 4 School of Psychology, Swansea University, Swansea SA2 8PP, UK; s.o.dymond@swansea.ac.uk; 5 Department of Psychology, Reykjavík University, 102 Reykjavík, Iceland; 6 Sussex Addiction Research and Intervention Centre and Sussex Neuroscience, University of Sussex, Brighton BN1 9RH, UK

**Keywords:** gambling, voluntary self-exclusion, contingency management

## Abstract

Research into self-directed methods for reducing problematic and harmful gambling is still in its infancy. One strategy that individuals use to prevent gambling involves voluntary self-exclusion (VSE) programs. For example, VSE programs can make it challenging to access betting sites or enable banks to block gambling-related transactions. Although individual VSEs can be helpful when used alone, it is unclear whether their efficacy is enhanced when combined. Furthermore, it is unknown how VSE compliance can be improved. We propose that contingency management (CM), an evidence-based strategy to incentivise abstinence, could encourage continued VSE use, promoting long-lasting recovery from problematic or harmful gambling. Here, we conducted exploratory analyses on VSE use and CM for gambling in two populations (members of the UK general population recruited and students). Participants responded favourably regarding combined VSE use. They felt that providing vouchers exchangeable for goods/services could incentivise gambling abstinence during VSE. However, some were concerned about people potentially “gaming” the system. Participants believed supplementing VSE and CM with social support could encourage abstinence. These attitudes, and recent research on treatment providers’ opinions on CM for gambling, suggest that experimental evidence should be sought to determine the efficacy of combined VSE use and CM for gambling.

## 1. Introduction

Continued gambling, despite experiencing harm, can be disruptive to individuals and their families. Harmful gambling is a growing public health concern [1,2,3] and is linked to other harms, such as substance misuse, depression, and suicidal ideation [4,5,6]. The growth of online gambling, expansion of the international gambling markets, and lack of unified legislation to tackle gambling across countries may explain why gambling may be a growing public health concern [7,8]. It is estimated that approximately one percent of the general population and eight percent of university students may meet the diagnostic criteria for problematic gambling [9,10]. Terminology related to gambling is evolving; here, we use problematic or harmful gambling (PG/HG) in an attempt to cover diverse experiences, incorporating both screening methods for problem gambling (e.g., using the Problem Gambling Severity Index, PGSI) and the experience of gambling harms (adverse impacts of gambling to the self, family, community and society, which are wide-ranging and challenging to measure) [11]. In this exploratory article, we aim to seek opinions on novel methods for reducing PG/HG and promoting long-lasting gambling abstinence.

Voluntary self-exclusion (VSE) from gambling may help combat betting urges and reduce gambling [12]. It is possible that effective VSE could mitigate high treatment drop-out rates in PG/HG [13]. VSE methods are opt-in programs that limit opportunities to gamble by blocking access to gambling, in-person or online, and vary across countries. For example, Sweden has a government-supported nationwide VSE (Spelpaus), which blocks access to licensed gambling in the country. Spelpaus enrolees are most likely to be users of online (casino, poker, and bingo) and land-based (casino, electronic gaming machine) types of gambling, and are less likely to be sports bettors [14].

In the UK, a variety of VSE methods are available which aim to block online gambling. For example, Gamban offers software-based VSE, which blocks access to gambling content on all devices on which it is installed [15]. Gamstop VSE prevents the registered user from accessing and creating accounts associated with gambling companies in the UK for up to 5 years [16]. Banks can also provide VSE when people request gambling-related ‘transaction blocks’ be placed on their accounts [17]. While VSE methods are often used individually, the UK Gambling Commission recently funded the TalkBanStop programme to support people’s simultaneous enrolment in Gamban, Gamstop, and Gamcare (for speaking with support advisors [18]) [19]. This manuscript, which presents data that was collected data before TalkBanStop was announced, investigates opinions on the combined use of multiple VSE methods.

We are particularly interested in student opinions of VSE methods. Student samples have indicated increased levels of stress and anxiety, which are understood to increase the risk of someone transitioning from at-risk to PG/HG [20]. Frequent exposure to gambling-related cues (especially while watching sporting events) [21,22], coupled with students’ increased stress and anxiety levels, may help explain why student populations show a higher prevalence of PG/HG compared to the general population [23,24]. Interestingly, others have found that the people registering for online self-exclusion were, on average, ten years younger than those registering for in-person gambling exclusion [25]. Thus, it may be that students find VSE methods especially attractive; we will assess this.

Some forms of PG/HG may be challenging to address by VSE methods; playing the national lottery may be one example. Given the wide availability of lottery tickets and because establishments do not always adhere to laws designed to help people self-exclude [12,26], it is unclear whether VSE methods could limit in-person lottery playing. That said, we did not find a relationship between playing the national lottery and PGSI score in UK students [24], and national lotteries often allow individuals to self-exclude from playing online [27]. There may also be issues with VSE methods reducing online gambling. Caillon et al. (2019) blocked access to participants’ favourite gambling websites for a week, which helped participants reduce their gambling in the short term but had no observable long-term effects [28]. Also, despite Spelpaus helping to reduce harm from gambling for many individuals, research has suggested that 38% of people in Sweden’s self-exclusion program were still gambling (e.g., through unregulated overseas online casinos and lotteries [14]); this is especially worrying, as offshore betting may be less-regulated and incur higher risks than nationally licensed establishments.

The limitations of employing a specific and short-term VSE to tackle a complex behavioural addiction are why some communities call for more far-reaching VSEs to be developed, perhaps in conjunction with behavioural therapies to comprehensively promote gambling abstinence [29]. However, it is also clear that some individuals enrolled in comprehensive VSEs may still be gambling despite wanting to stop [14]. Consequently, effective self-exclusion may involve supplementation with behavioural therapies, further promoting gambling abstinence.

One potential behavioural therapy is contingency management (CM). Developed for treating substance use disorders, CM incentivises specific behaviours by rewarding abstinence or other recovery-related target behaviours [30,31]. For example, during CM, increasing reward values (e.g., vouchers for services) promote continued abstinence; if an individual relapses, the reward amount is reset to its initial value [32]. Because similar neurobiological processes may underlie substance misuse and harmful gambling [33], it may be that providing alternative rewards via CM could help people reduce their gambling. Recent UK studies have assessed clients’ and gambling treatment service providers’ views on CM and validated a scale to measure the same; while providers were somewhat receptive to CM for gambling (if it was demonstrated effective), they shared various concerns, ranging from CM reinforcing similar behaviours to PG/HG, to decreasing trust with people seeking help for their harmful gambling [34,35,36]. That said, similar concerns are often voiced regarding CM for substance misuse [31]. It is possible that practitioner-perceived similarities between CM and gambling may be a benefit of CM, where unhealthy PG/HG behaviours can be substituted with controlled CM reinforcement. It is worth further understanding perceptions of CM for gambling to determine the likelihood of its future adoption.

The present research is made up of two studies that assess (a) the public and (b) university student opinions of VSE methods (Gamban, Gamstop, and bank transaction blocks) and how their use can be combined. Furthermore, in these populations, we surveyed opinions on how providing rewards (via CM) may promote adherence to VSE. Accordingly, participants were asked how to implement CM most effectively, as well as how discussions to identify adherence to self-exclusion should take place to support gambling self-exclusion and reduce gambling harms.

## 2. Methods and Procedure

### 2.1. Participants

#### 2.1.1. Study 1 (S1): Prolific

Participants were recruited through Prolific, an online platform used for survey distributions. A total of 95 responses were received; 11 incomplete responses were removed, resulting in the responses of 84 participants being available for analysis. Participants were aged 18 or over, with slightly more participants identifying as female (54.76%) than male (45.24%). Participants received £3.25 on completion of the study. All participants were debriefed and provided with sources of further support. The Research Ethics Committee at the University of Sussex approved this study (ER/MZ285/3).

#### 2.1.2. Study 2 (S2): Students

An opportunity sample of UK university students was recruited via online and social media distribution. A total of 63 responses were received; 12 incomplete responses were removed, resulting in 51 responses being available for analysis. Participants were aged between 20 and 33 years old (16 identified as male, 34 as female, and 1 as non-binary). Unlike in Study 1, participants were not compensated for their time. All participants were debriefed and provided with sources of further support. This study was approved by the Research Ethics Committee at the University of Sussex (ER/MZ285/5).

### 2.2. Measures

All participants answered questions via the Qualtrics online survey platform. Participants were asked for demographic information, followed by their opinions on how three VSE methods (Gamban, Gamstop, and transaction blocks) could be used individually or in combination to reduce PG/HG. Participants were also asked whether CM could support gambling self-exclusion, and how this might be achieved.

Bespoke questions, adapted from the Problem Gambling Severity Index (PGSI), were used to unpack VSEs’ perceived abilities to reduce PG/HG. Example questions included ‘I would encourage someone/myself to install Gamban if I were concerned about their/my own well-being’, ‘If gambling was causing health problems, including stress or anxiety, singing up to Gamstop would reduce these health problems’, and ‘Bank gambling-transaction blocks would stop me/someone from gambling, even if I/they didn’t think that they could’. Participants rated their agreement with these statements on a scale from 1 (extremely disagree) to 7 (extremely agree). When answering these questions, participants were asked to reflect on their gambling or someone else’s.

Participants were asked several questions regarding the use of CM for gambling. Numerical values were recorded for the minimum and maximum weekly and total voucher earnings thought to be effective for CM, as well as the amount of time to achieve abstinence via CM. Individuals were asked to comment on the amounts they chose; example quotes are displayed in the results section. We also asked what type of voucher participants would prefer (for supermarkets, e-commerce, experiences, or high-street retailers).

Finally, we asked a series of questions relating to with whom the participants would want to discuss gambling, the use of VSE, and adherence to CM. Questions explored discussion preferences, comfort levels, least distressing, and the promotion of honesty. Each question was rated on a scale of 1 to 7, indicating preferences for discussing gambling in a group versus one-on-one with another individual. Participants indicated the person they would be willing to discuss their gambling with (multiple options could be chosen: general practitioner (GP), psychiatrist/clinical psychologist, social worker, sponsor, community member, family member, friend, stranger, or no one). Individuals were allowed to comment on their choices.

### 2.3. Data Analysis

All data analysis was performed using SPSS (version 28). Friedman’s tests and post hoc analyses were used to analyse responses to the bespoke questions. Chi-square tests were used to analyse the frequencies of the most/least favourable VSE method, as well as the most/least favourable VSE combinations. Descriptive statistics were used to analyse preference towards discussing gambling one-to-one, as well as for which individual was preferred to discuss gambling with. Chi-square tests were used to assess participants’ preference towards which incentive to use in CM.

## 3. Results

The present study aimed to assess opinions on strategies to improve the success of gambling self-exclusion. We assessed views on combined enrolment in multiple self-exclusion programs instead of taking part in a single method. We also asked whether people think problematic gambling can be reduced by rewarding adherence to self-exclusion programs. Additionally, we assessed public opinion on whether specialist-guided or community-based therapies or conversations about gambling can be used to improve self-exclusion from gambling. Crucially, these results are exploratory, representing opinions on VSEs programs and CM in general populations, in people who may or may not gamble; we hope that these findings encourage and guide future work on these strategies, which may help promote long-lasting recovery from PG/HG.

### 3.1. Assessment of Self-Exclusion Methods

#### 3.1.1. How Are Individual VSE Methods Perceived?

People believed that all the VSEs studied (Gamstop, Gamban, and bank transaction blocks) would be effective in reducing gambling. However, gambling-related transaction blocks were often perceived to be the best at reducing aspects of PG/HG. Friedman’s statistical tests were used to compare scores given for the three different self-exclusion methods on each of the ‘bespoke’ questions (scale from one, extremely disagree, to seven, extremely agree; reported results refer to means (M) and standard deviations (SD)). While Study 1 (S1; Prolific) and Study 2 (S2; Student) are reported separately, they produced similar findings.

We first asked whether the individual would encourage someone to register for each VSE method if they were worried about the individual’s gambling. Participants encouraged the use of each VSE method: Gamstop (S1, M = 5.95, SD = 1.33; S2, M = 5.47, SD = 1.48), Gamban (S1, M = 5.69, SD = 1.54; S2, M = 5.47, SD = 1.61), and transaction blocks (S1, M = 6.20, SD = 1.20; S2, M = 6.02, SD = 1.21). There was a significant difference in support between the VSE method (S1, X^2^(2) = 13.34, *p* ≤ 0.001. S2, X^2^(2) = 13.57, *p* < 0.001), with banking transaction blocks being the most highly encouraged (post hoc tests: S1, transaction blocks vs. Gamban, *p* = 0.011; S2, transaction blocks vs. Gamstop, *p* = 0.012).

Similar results were found in the perceived effectiveness of the three VSE methods for helping someone stop gambling, even if they did not think that they could stop. While Gamstop (S1, M = 5.20, SD = 1.56; S2, M = 4.55, SD = 1.61), Gamban (S1, M = 4.78, SD = 1.53; S2, M = 4.19, SD = 1.46), and transaction blocks (S1, M = 5.24, SD = 1.75; S2, M = 5.22, SD = 1.49) were all considered to be effective, there were significant differences in this measure (S1, X^2^(2)= 10.41, *p* = 0.005. S2, X^2^(2) = 14.85, *p* = 0.001). For Study 1 (Prolific), Gamstop and transaction blocks appeared equally effective (post hoc tests: transaction blocks vs. Gamban, *p* = 0.037. Gamstop vs. Gamban, *p* = 0.043). Students perceived transaction blocks to be most effective in Study 2 (post hoc tests: transaction blocks vs. Gamban, *p* = 0.001).

We next asked whether each of the VSE methods would be effective at reducing health problems (e.g., stress or anxiety), and we again found that each VSE was considered effective: Gamstop (S1, M = 5.22, SD = 1.50), (S2, M = 4.52, SD = 1.37), Gamban (S1, M = 5.04, SD = 1.53; S2, M = 4.30, SD = 1.49), and transaction blocks (S1, M = 5.44, SD = 1.54; S2, M = 5.05, SD = 1.52). In particular, banking transaction blocks were perceived as being highly effective in reducing gambling-related health problems (S1, X^2^(2) = 9.70, *p* = 0.008. vs. Gamban, *p* = 0.025. S2, X^2^(2) = 9.48, *p* = 0.009. vs. Gamstop, *p* < 0.001, or Gamban, *p* = 0.046).

Finally, two questions addressed the efficacy of VSE methods for ameliorating finance-related issues. The perceived effectiveness of the three VSEs at stopping someone from borrowing or selling possessions in order to gamble significantly differed significantly (S1, X^2^(2) = 16.66, *p* < 0.001. X^2^(2) = 21.60, *p* < 0.001). Again, all VSEs were considered effective for reducing borrowing/stealing [Gamstop (S1, M = 4.94, SD = 1.71; S2, M = 4.28, SD = 1.37), Gamban (S1, M = 4.39, SD = 1.82; S2, M = 4.02, SD = 1.54), and transaction blocks (S1, M = 4.82, SD = 1.90; S2, M = 4.99, SD = 1.69)]. Post hoc tests revealed slight differences between our Prolific (Gamban vs. Gamstop, *p* = 0.002; Gamban vs. transaction blocks, *p* = 0.028) and student (transaction blocks vs. Gamban *p* < 0.001 or Gamstop *p* = 0.046) populations. Lastly, all VSEs were recommended for reducing financial burdens [Gamstop (S1, M = 5.99, SD = 1.29; S2, M = 5.22, SD = 1.45), Gamban (S1, M = 5.99, SD = 1.53; S2, M = 5.19, SD = 1.58), and transaction blocks (S1, M = 6.08, SD = 1.28; S2, M = 5.80, SD = 1.39)]. There may have been a ceiling effect in Study 1, as there were no significant differences between the VSEs (X^2^ = 0.41, *p* = 0.817). In contrast, for Study 2, students did report a difference in the perceived efficacy of the VSEs for helping households with financial difficulties (X^2^(2) = 8.77, *p* = 0.012; transaction blocks vs. Gamban, *p* = 0.001).

#### 3.1.2. What Combinations of VSE Methods May Be Considered Beneficial?

According to our assessment of individual VSE methods, bank transaction blocks are often considered the most effective for helping to reduce problematic gambling. Crucially, individuals seeking to reduce their gambling can register with multiple VSEs. Participants were asked to rank combinations of VSE methods in order of their perceived efficacy for helping someone to stop gambling. As shown in Table 1, Chi-square goodness of fit tests revealed that, for both Study 1 (Prolific) and Study 2 (Student), participants thought combining bank transaction blocks, Gamstop, and Gamban was the most effective way of decreasing gambling. Following this three-VSE approach, the combined use of transaction blocks with either Gamban or Gamstop was considered equally effective. In contrast, the least effective combination lacked transaction blocks for both studies.

### 3.2. Opinions on Using CM Therapy to Support Self-Exclusion

#### 3.2.1. How Might CM for Gambling Be Delivered?

Participants generally supported CM for PG/HG and provided insights into how a CM program might be designed. Participants from both studies thought that delivering a financially based reward could incentivise someone to stop gambling [rated on a scale of 0 (very unhelpful) to 7 (very helpful); S1, 4.99 (1.70); S2, 4.45 (1.34)]. Participants were then asked what types of rewards should be used. Differences were observed in the preferred form of reward for CM for Study 1 (Prolific; x^2^(3) = 14.66, *p* = 0.002) but not Study 2 (student; x^2^(3) = 7.16, *p* = 0.067). In general, vouchers for e-commerce (e.g., Amazon vouchers) were the most favourable (S1, n = 54; S2, n = 30), followed by vouchers for supermarkets (S1, n = 48; S2, n = 20), then vouchers for experiences (e.g., restaurants or cinema; S1, n = 32; S2, n = 26), with vouchers for major in-person shopping retailers (e.g., clothing stores; S1, n = 24; S2, n = 13) being the least favourable. The selection of voucher type was not mutually exclusive (i.e., participants could pick all voucher types that they thought would help incentivise gambling).

Participants believed the CM program should last approximately seven weeks (S1, M = 7.54, SD = 2.65; S2, M = 6.47, SD = 2.36). The survey provided text boxes where participants could explain why they chose a given length for CM to be effective. While several individuals indicated that there would be individual variation in the amount of time required, a common comment related to more extended CM periods being needed to break a ‘gambling habit.’ Some, however, were worried that an extended length of CM might ‘incite fraud to make the scheme too inviting by making it a viable source of income,’ although this was countered by others suggesting that lengthy CM can help people ‘get out of poverty and change their lives’.

Table 2 describes the minimum and maximum weekly and total amounts of voucher-based reinforcement participants believed were effective/appropriate for CM. In general, higher voucher values were recorded by Prolific participants (S1) than students (S2); such differences could reflect various participant characteristics (e.g., age, social, housing, and employment differences; motivation to pursue monetary reward, since only participants in the Prolific cohort were compensated). According to comments left on the survey, monetary values were chosen for several common reasons. For example, some individuals compared the voucher value to the amount they would typically try to win when betting; therefore, they required the voucher to be similar or greater in value to incentivise them to stop gambling (e.g., ‘It is close to the amount I often spend on a weekly basis when gambling’). For other individuals, the voucher value needed to be for an amount that would enable them to achieve a particular goal (e.g., ‘I thought about what would be enough to buy a substantial treat on Amazon, enough to persuade me not to place any bets’ or ‘£25 a week building up quickly which would encourage me to keep going [in CM]. If I opted for the supermarket vouchers too, it would cover a considerable amount of my weekly food spend’). Other individuals were worried that vouchers with excessive monetary values could be problematic (e.g., ‘Too much money could become addictive in itself’ and ‘encourage fraudulent behaviour’).

#### 3.2.2. How Might Social Support Facilitate CM for Gambling?

The success of CM for gambling may require individuals to discuss their behaviour (and use of VSEs) with people who support them. Participants’ opinions indicated consistent preferences towards discussing gambling one-on-one with another individual instead of in a group setting for both studies (Table 3). One participant described not wanting to participate in a group because there may be a ‘temptation [for gamblers] to outdo each other’.

People preferred to discuss gambling with a mental health professional, such as a psychiatrist or clinical psychologist (Table 4). Participants explained why they felt this, for example, ‘It is really embarrassing to talk about [gambling] and many people judge or do not understand. A trained professional is definitely needed.’ Another participant stated, ‘I think “keeping face” is very important for gamblers. It is quite easy to have a gambling addiction that is completely invisible to your closest friends and family… it may be liberating to feel that they can solve this problem without risking their reputation further by bringing their addiction to light’. Other participants also expressed the challenges of discussing gambling with relatives: ‘Admitting gambling habits to others feels almost impossible to me. The thought of my family or partner knowing frightens me, so I am secretive and thus more likely to relapse as it is only me regulating my problem’.

Interestingly, for the Prolific study, a sponsor was the second most popular choice, while students preferred to speak with a friend; such differences could relate to perceived differences regarding abstinence programs across groups. Several individuals highlighted how honesty is essential to discussions regarding gambling, with a need to ‘build up an honest, safe and meaningful relationship’, and that this might be achieved by developing a supportive relationship with a sponsor or mental health professional.

## 4. Discussion

The present study assessed the general public’s (Study 1) and university students’ (Study 2) opinions on three current VSE methods and thoughts on combined enrolment in multiple VSEs. Furthermore, participants’ thoughts on whether PG/HG could be reduced by supporting self-exclusion with CM, and opinions on how to most effectively implement CM to support self-exclusion, were assessed. Similar results were observed in both groups of participants, with support for all three VSE methods (Gamstop, Gamban, and bank transaction blocks) and enthusiasm for the use of CM to promote gambling abstinence (supplemented with one-on-one support).

### 4.1. Comparison of VSE Methods

The present study revealed that all VSE methods studied were received positively as a strategy to reduce PG/HG. Transaction blocks were perceived to be the most favourable at reducing behaviours associated with PG/HG, as well as the perceived most effective at stopping someone from gambling. Moreover, participants felt that gambling abstinence was most likely to be achieved when all three VSE methods were used in combination.

The favourability of banking transaction blocks may be explained by their simplicity, only needing to engage with an organisation they already have a relationship with (i.e., their bank), thereby counteracting the lack of treatment-seeking behaviours among individuals with PG/HG [37]. Transaction blocks may also be popular because they can help prevent both online and in-person gambling. However, such blocks are limited in that not all banks provide them, in which case, an individual would need to sign up for another available VSE method (e.g., Gamstop or Gamban).

It is still unclear how effective VSE is in practice at reducing gambling. Hâkansson and Widinghoff (2020) found that 38% of people enrolled in comprehensive Swedish VSE were still gambling [14]. Furthermore, the VSE methods researched in this study may be limited in their effectiveness at reducing gambling via lottery tickets. For example, Zolkwer and colleagues (2022) found that the most reported form of gambling among UK university students was via lottery tickets [24]. That being said, online sports betting was also a popular activity amongst students [24], which could be reduced by VSE. VSE methods may also struggle to regulate gambling-like content within video games, which could influence the development of offline PG/HG [38,39,40]. Because of vast individual variation and the variety of gambling experiences available, the reasons why specific individuals may benefit from VSE still require significant investigation [41,42,43,44].

Individuals experiencing PG/HG may learn about VSEs from gambling operators, whom governmental agencies may require signposting support mechanisms. Over the past few years, there has been an increase in gambling operators supplying such information; however, methods are often advertised for setting limits or ‘taking a break’ from gambling rather than signing up for software that blocks gambling altogether, such as Gamban [45]. Crucially, ‘taking a break’ via short-term self-exclusion is less effective than long-term self-exclusion at reducing future gambling [46].

The UK Gambling Commission recently funded TalkBanStop, a compound VSE-based intervention for PG/HG [19]. While there is scant scientific research on the efficacy of combining VSE methods, our data, and the enthusiasm for TalkBanStop, illustrate the potential for this therapeutic approach. While our study did not explicitly ask whether participants would desire the guidance of a Gamcare advisor (an effective method of help for gamblers [18]), we demonstrated that social support may be helpful during the VSE- and CM-mediated journey to abstinence, especially from someone who has expertise in helping individuals who experience PG/HG.

Given that infringements in gambling self-exclusion occur [12,14], it is crucial to develop methods to maximise effectiveness at reducing harms from, and access to, gambling. In Ontario, a voluntary self-exclusion program involves treatment-seeking individuals signing a contract preventing access to gambling venues; breach of this contract may result in fines and criminal charges for trespassing [26]. While this may be helpful, there are potential issues with the program, including gambling operators not complying (i.e., allowing self-excluded individuals into the casino) and ethical concerns regarding potentially vulnerable individuals (including those with a diagnosis of gambling disorder) signing a legal contract related to their mental wellbeing [26]. According to instrumental/operant conditioning, such programs can be considered a form of response-cost punishment, where an action (e.g., entering a casino) has negative consequences (e.g., a fine), which would decrease the likelihood of the individual attempting to gamble. In contrast, CM for gambling is a type of behavioural positive reinforcement, where a chosen action (e.g., not entering a casino) is rewarded (e.g., by receiving a voucher), thereby encouraging the action (i.e., the decision not to gamble and subsequent abstinence). Such different VSE approaches may not be mutually exclusive and could be combined; this may be a topic of future clinical studies.

### 4.2. CM for Gambling

Shared biomedical and psychosocial underpinnings of substance use disorder and PG/HG suggest that they may be treated with similar forms of therapy [33,47]. Despite this, CM, one of the most successful treatments for substance use disorders (upwards of 61% drug-free success rates for CM vs. 39% for control groups), has not been established as a possible therapy for PG/HG [30,48,49]. CM protocols reward participants with monetary vouchers when they successfully pass a drug test (e.g., urine samples that screen negative for an abused substance); based on the results of this manuscript, we believe it may be possible to incorporate VSE methods into a CM program for PG/HG. Clinical trials and pilot experiments that use CM to treat gambling have only recently been proposed [50,51].

CM for gambling has recently been examined from a treatment provider’s perspective [34], and the results were similar to those reported in this manuscript; both potential service users and providers are interested in the idea of CM for gambling. Our explanatory analysis of CM procedures provides basic guidance for implementing CM therapy to support self-exclusion, where the gambling discussion is one-to-one between the client and therapist. This finding supports Nuske and Hing’s research (2013), which found that the stigma surrounding gambling was potentially exacerbated in a group setting, potentially blocking treatment-seeking [52].

The present studies only asked participants about their perceptions towards financially incentivising gambling abstinence. While this was received positively, it is a controversial idea [31]. Indeed, practitioners may prefer incentivising specific treatment goals or attendance rather than abstinence to improve treatment outcomes for those experiencing PG/HG [34]. Future research could investigate perceptions of using CM to achieve these objectives in treatment-seeking individuals [36].

Participants varied in the type of voucher they preferred to receive as part of CM. Therefore, like other forms of therapy to reduce PG/HG, treatment methods such as choosing a particular incentive should be discussed with each client [53]. Similarly, the amount and duration of reinforcement during CM should accommodate individual needs and vary according to gambling severity. Providers of CM for gambling may also feel the need to monitor clients closely, as some practitioners may think that such a program could trigger relapse or that patients may take advantage of CM to gain resources for gambling (e.g., vouchers sold as cash) [35].

### 4.3. Limitations and Future Work

In general, the findings of Study 1 (Prolific recruitment) and Study 2 (student recruitment) were similar. Opinions on the VSE methods were comparable, although there was some indication that students would be more likely to encourage someone to register for an application like Gamban. Students (Study 1) were also more likely to recommend smaller voucher amounts than individuals in the Prolific study. Furthermore, students were likelier to see a GP and less likely to access a sponsor (e.g., via Gamblers Anonymous) to discuss their gambling concerns. While such results may suggest an age- or education-related shift in where individuals seek care for PG/HG, more research is needed to explore this. Together, while these observations are interesting, they are descriptive and not statistically analysed. We did not create a complete demographic profile of participants in either study, which makes comparison challenging (e.g., we do not know if some participants in the Prolific study are also students). Crucially, sample sizes for both studies were relatively small (Study 1, n = 95; Study 2, n = 63; limited by funding and timing). Regardless, the two studies produced subjectively similar results, suggesting support for their validity/replicability.

Future research could look to use a more concentrated sample of gamblers. Or, perhaps rather than gambling motivations, the relationship between comorbid psychiatric conditions such as problem drinking and depression and thoughts on CM and VSE could be analysed [54]. Understanding how the experience of these comorbidities affects thoughts on both CM and VSE methods could help achieve the goal of understanding who is most amenable to these interventions and who may require extra support with self-exclusion. To support this, future work may investigate how CM and VSE can be included as potential programs of support via expanded screening for PG/HG in health and social care settings.

### 4.4. Conclusions

The original purpose of this research was to explore how VSE methods, individually or in combination, could be used as part of a CM program for PG/HG. While such an application requires much further development, it is possible that automated assessment of adherence to VSE, when combined with social support (e.g., one-on-one guidance from a mental health professional), could provide the foundation of a voucher-based program to incentivise gambling abstinence or motivating attainment of other treatment goals. Overall, across two studies, participants shared positive attitudes on VSE methods for reducing gambling and the viability of CM to help those who experience PG/HG. Participants shared concerns about CM promoting gambling or fraud-related activities for some individuals, and such worries have been previously highlighted by practitioners [34,35]. Along with the support voiced for CM, such apprehensions should be addressed in future work that assesses the viability of incorporating VSE methods into a CM strategy for PG/HG tailored to individual needs.

## Figures and Tables

**Table 1:** Frequencies and Chi-square statistics for perceived most effective to least effective combinations of VSE methods for Studies 1 (Prolific) and 2 (Student).

	Most Effective		Least Effective	
	Study 1	Study 2	Study 1	Study 2
Transaction blocks, Gamstop, Gamban	53	33	4	4
Gamban and transaction blocks	16	7	6	8
Gamstop and transaction blocks	10	7	10	7
Gamban and Gamstop	5	4	62	32
x^2^	67.91	43.35	108.38	39.43
*p*	<0.001	<0.001	<0.001	<0.001

**Table 2:** Descriptive statistics for maximum and minimum CM values and mean (SD).

	Study 1	Study 2
Weekly Amount	Min: £31.34 (17.07) Max: £81.43 (56.94)	Min: £24.70 (15.90) Max: £71.16 (38.96)
Total Amount	Min: £282.84 (201.69) Max: £422.03 (209.95)	Min: £217.66 (147.00) Max: £367.00 (196.59)

**Table 3:** Study 1 (Prolific): Descriptive statistics for the participant responses to questions regarding discussing gambling behaviour in a group (1) or one-to-one (7).

Preference and Reason for Discussing Gambling in a Group (1) or One-to-One (7)	Study 1—Prolific Mean (SD)	Study 2—Student Mean (SD)
Prefer	5.07 (2.04)	4.47 (2.12)
More comfortable	5.27 (1.94)	4.63 (1.92)
Less distressing	5.33 (1.89)	4.47 (2.04)
Promote honest discussion	4.93 (2.03)	4.89 (1.96)

**Table 4:** Percent of respondents indicating their preferred individual to discuss gambling with.

	Study 1 (Prolific)	Study 2 (Student)
Community Member	5%	7%
Family Member	9%	8%
Friend	14%	16%
GP	8%	14%
Psychiatrist or Psychologist	27%	29%
Social Worker	7%	8%
Sponsor	21%	8%
Stranger	9%	8%
No One	0%	2%

## Data Availability

The data is available upon request.
